# Evaluation of Changes in Activities of Daily Living and Quality of Life of Patients with Bone Metastasis Who Underwent Conservative Therapy through Bone Metastasis Cancer Boards

**DOI:** 10.3390/medicina60060906

**Published:** 2024-05-29

**Authors:** Yasumitsu Fujii, Ryo Yoshikawa, Ryoga Kashima, Wataru Saho, Hirokazu Onishi, Tsuyoshi Matsumoto, Risa Harada, Yoshiki Takeoka, Ryoko Sawada, Naomasa Fukase, Hitomi Hara, Kenichiro Kakutani, Toshihiro Akisue, Yoshitada Sakai

**Affiliations:** 1 Department of Physical Medicine and Rehabilitation, Kobe University Hospital 7-5-2, Kusunoki-cho, Chuo-ku, Kobe 650-0017, Hyogo, Japanyossie@med.kobe-u.ac.jp (Y.S.); 2 Division of Rehabilitation Medicine, Kobe University Graduate School of Medicine 7-5-2, Kusunoki-cho, Chuo-ku, Kobe 650-0017, Hyogo, Japan; 3 Department of Orthopaedic Surgery, Kobe University Graduate School of Medicine 7-5-1, Kusunoki-cho, Chuo-ku, Kobe 650-0017, Hyogo, Japan; 4 Department of Rehabilitation Science, Kobe University Graduate School of Health Sciences 7-10-2, Tomogaoka, Suma-ku, Kobe 654-0142, Hyogo, Japan; akisue@med.kobe-u.ac.jp

**Keywords:** bone metastasis, ADL, QOL, cancer board, multidisciplinary treatment

## Abstract

*Background and Objectives*: Changes in activities of daily living (ADL) and quality of life (QOL) of patients with bone metastasis who underwent surgical treatment through Bone Metastasis Cancer Boards (BMCBs), a recent multidisciplinary approach for managing bone metastases, have been reported; however, no reports exist on patients who undergo conservative treatment. In this study, we aimed to evaluate these patients’ ADL and QOL and examine the factors influencing changes in these parameters. *Materials and Methods*: We retrospectively reviewed 200 patients with bone metastases who underwent conservative therapy through BMCBs between 2013 and 2021. A reassessment was conducted within 2–8 weeks after the initial assessment. Patients’ background and changes in performance status (PS), Barthel Index (BI), EuroQol five-dimension (EQ-5D) scores, and Numerical Rating Scale (NRS) scores were initially assessed. Furthermore, we categorized patients into two groups based on improvements or deteriorations in ADL and QOL and performed comparative analyses. *Results*: Significant improvements in EQ-5D (0.57 ± 0.02 versus [vs.] 0.64 ± 0.02), NRS max (5.21 ± 0.24 vs. 3.56 ± 0.21), and NRS average (2.98 ± 0.18 vs. 1.85 ± 0.13) scores were observed between the initial assessment and reassessment (all *p* < 0.001). PS (1.84 ± 0.08 vs. 1.72 ± 0.08) and BI (83.15 ± 1.68 vs. 84.42 ± 1.73) also showed improvements (*p* = 0.06, and 0.054, respectively). In addition, spinal cord paralysis (odds ratio [OR]: 3.69, *p* = 0.049; OR: 8.42, *p* < 0.001), chemotherapy (OR: 0.43, *p* = 0.02; OR: 0.25, *p* = 0.007), and NRS average scores (OR: 0.38, *p* = 0.02; OR: 0.14, *p* < 0.001) were independent factors associated with ADL and QOL. *Conclusions*: Patients with bone metastases who underwent conservative treatment through BMCBs exhibited an increase in QOL without a decline in ADL. The presence of spinal cord paralysis, absence of chemotherapy, and poor pain control were associated with a higher risk of deterioration in ADL and QOL.

## 1. Introduction

Owing to advances in cancer treatment, the number of survivors of cancer has been increasing, with an estimated 5.33 million survivors in 2015. With the increase in the number of patients with cancer, the number of patients with bone metastases has also been increasing rapidly [1]. Bone is the third most common site of metastasis, following the lungs and liver; therefore, it ranks among the most frequent sites of metastatic disease [2,3]. Bone metastasis occurs in approximately 5–10% of all patients with cancer. The primary lesion sites are often lungs, breasts, prostate, and kidneys [4], whereas the spine is the most common metastatic site, followed by the femur, pelvis, and ribs [5,6].

One of the most severe complications of bone metastasis is skeletal-related events (SREs), which include pathological fractures that can impair ambulation and vertebral compression or fractures that induce spinal cord compression, resulting in numbness, weakness, urinary/fecal incontinence or spinal cord paralysis [7]. SREs can significantly impair the Activities of Daily Living (ADL) and Quality of Life (QOL) of patients with cancer and greatly impact the prognosis [8]. Notably, SREs such as pathological fractures of long bones, spinal cord paralysis caused by spinal cord compression, and hypercalcemia require urgent treatment [9]. Therefore, the prevention and early detection of SREs are crucial to the management of bone metastases.

Bone metastases are often difficult to cure, and the treatment goals often include improving or maintaining patients’ performance status (PS), ADL, and QOL [10]. A multidisciplinary approach from various perspectives is required for the management of bone metastases, which requires interdisciplinary and collaborative treatment from multiple medical specialties. The effectiveness of interdisciplinary treatment has been reported in several studies [11,12]. Furthermore, one of the most important challenges of bone metastasis is its destruction, which results in the need for materials with bone regeneration and anti-cancer properties [13]. To achieve interdisciplinary treatment, it is crucial to hold meetings involving multiple medical specialties to exchange opinions and determine accurate diagnosis and treatment strategies [14]. Recently, the Bone Metastasis Cancer Board (BMCB), an initiative in which treatment strategies for patients with bone metastasis are discussed by a multidisciplinary team from various medical specialties, has been reported. We have been implementing this initiative since 2013. Changes in ADL and QOL of patients with bone metastasis who underwent surgical treatment through the BMCB have been reported [15,16]; however, no reports exist on patients who undergo conservative treatment. In this study, we targeted cases where surgical treatment was not indicated by the BMCB, metastases were not observed in sites requiring surgery, or where surgery was not desired by the patients or their families. The objective was to demonstrate the importance of conservative therapy through the BMCB for patients with bone metastasis, even without surgical intervention. Therefore, this study aimed to investigate changes in ADL and QOL among patients with bone metastasis and examine the factors influencing these changes.

## 2. Materials and Methods

### 2.1. Ethics Statement

This study was approved by the relevant institutional review boards (IRBs) of Kobe University Hospital, Japan (IRB number: B210055). Written informed consent was obtained from each patient according to the principles outlined in the Helsinki Declaration regarding human research.

### 2.2. Participants and Data Collection

**Figure 1:**
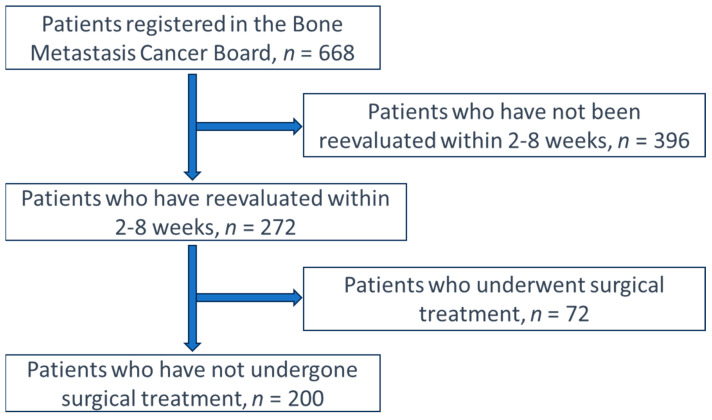
Selection process of patients in the study.

In this study, among the 668 individuals who were eligible for treatment through the BMCB at our facility between January 2013 and January 2021, the ADL and QOL of 272 (40.7%) were reassessed within 2–8 weeks after the initial assessment. Among these, 200 individuals who underwent conservative therapy were finally included in this analysis (Figure 1). To confirm the extent of bone metastasis, all patients underwent radiography, computed tomography (CT), and/or magnetic resonance imaging (MRI). Remote metastases were evaluated as required using (enhanced) CT/MRI, bone scans, and positron emission tomography with 18F-fluoro-2-deoxy-D-glucose.

We obtained clinical data from our database. These included information on patients’ background: age and sex; tumor progression: the presence of primary lesions, as classified by Katagiri [15], and visceral/brain metastasis or dissemination; treatment: history of rehabilitation, chemotherapy, radiotherapy, and the use of a bone–modifying agent; and ADL: Eastern Cooperative Oncology Group Performance Status. Regarding bone metastases, we examined the number and localization (spine, femur, pelvis, or others). In this study, an SRE was defined as a pathological fracture, spinal cord paralysis, and hypercalcemia.

PS, ADL, QOL, and pain (maximum [max] and average values) were initially assessed and reassessed within 2–8 weeks after the initial assessment. ADL, QOL, and pain were assessed using the Barthel Index (BI), Euro-QoL five-dimension (EQ-5D) scores, and Numerical Rating Scale (NRS) max and average scores, respectively. To examine the factors influencing changes in ADL, we divided the patients into two groups based on increased BI (“Ia” group) or declined BI (“Da” group) for comparison. Similarly, to examine the factors influencing changes in QOL, we divided the patients into two groups based on increased EQ-5D scores (“Iq” group) or declined EQ-5D scores (“Dq” group) for comparison.

### 2.3. Statistical Analyses

Statistical analysis was performed using IBM SPSS Statistics for Windows (version 26; IBM, Armonk, NY, USA). We performed a Mann–Whitney U test to examine factors associated with changes in the assessment scores. Subsequently, we evaluated factors influencing the BI and EQ-5D scores using univariate analysis with a χ² test. We performed a multivariate logistic regression analysis (using the stepwise method with likelihood ratio) to explore independent factors associated with improvements in ADL and QOL. Statistical significance was set at *p* < 0.05.

## 3. Results

Table 1a presents patients’ demographics. The mean duration from the initial assessment to the reassessment was 33.81 ± 0.86 days. The average age of the patients was 72.2 ± 12.9 years. There were 116 males (58%) and 84 females (42%). Tumor progression was classified according to Katagiri classification: slow growth (*n* = 48 [24%]), moderate growth (*n* = 71 [35.5%]), and rapid growth (*n* = 81 [40.5%]). Regarding sites of metastases other than the bone, 113 patients (56.5%) had no metastasis, 77 (38.5%) had visceral or brain metastasis, and 10 (5%) had disseminated metastasis.

Sixty-nine patients (34.5%) underwent rehabilitation therapy, 118 (59%) underwent chemotherapy, 104 (52%) underwent radiotherapy, and 79 (39.5%) received bone-modifying agents. Among the patients, 133 (66.5%) had multiple bone metastases. The most common site of bone metastasis was the spine (*n* = 177 [88.5%], followed by the femur/pelvis (*n* = 54 [27%]), and others (*n* = 42 [21%]).

Regarding SREs, 21 patients (10.5%) developed spinal cord paralysis at the initial assessment, 23 (11.5%) presented with pathological fractures during the evaluation period, and 19 (9.5%) developed hypercalcemia. Table 1b presents the distribution of primary tumors. Lung cancer (17.3%) was the most common primary tumor, followed by renal cell carcinoma (12.1%) and head and neck cancer (7.7%).

Table 2 presents the mean BI and PS, EQ-5D, NRS max, and NRS average scores at the initial assessment and reassessment. Significant improvements (initial assessment versus [vs.] reassessment) in EQ-5D (0.57 ± 0.02 vs. 0.64 ± 0.02), NRS max (5.21 ± 0.24 vs. 3.56 ± 0.21), and NRS average (2.98 ± 0.18 vs. 1.85 ± 0.13) was observed between the initial assessment and reassessment (all *p* < 0.001). In addition, PS (1.84 ± 0.08 vs. 1.72 ± 0.08) and BI (83.15 ± 1.68 vs. 84.42 ± 1.73) showed a trend towards improvement, although no statistically significant differences were observed (*p* = 0.065 and 0.054, respectively).

To investigate the factors influencing changes in ADL, the patients were divided into Ia and Da groups. Table 3a presents patients’ characteristics in the two groups. Regarding primary lesions, the incidence of slow growth was significantly higher in the Ia group, whereas that of rapid growth was significantly higher in the Da group (*p* < 0.05). The prevalence of pathological fractures, spinal cord paralysis, and absence of chemotherapy were significantly higher in the Da group (*p* < 0.001). The proportion of patients with improved NRS max and average scores was significantly higher in the Ia group (*p* = 0.020 and 0.004., respectively).

To further explore the independent factors associated with improvement in ADL, we performed a multivariate logistic regression analysis (using a forward stepwise method: likelihood ratio). The dependent variable was indicated as 0 for the group with improved ADL and 1 for that with deteriorated ADL. Independent variables included in the analysis were selected from Table 3a based on a significance level of *p* < 0. Theynd were primary lesions, as classified by Katagiri, the presence of pathological fractures, spinal cord paralysis, chemotherapy, radiotherapy, changes in NRS scores (average and max), age, and sex. The degree of primary tumor progression (OR: 1.731, *p* = 0.032), spinal cord paralysis (OR: 3.693, *p* = 0.049), chemotherapy (OR: 0.429, *p* = 0.02), and NRS average scores (OR: 0.378, *p* = 0.02) were found to influence ADL deterioration (Table 3b).

To investigate the factors influencing changes in QOL, we divided the patients into Iq and Dq groups. Table 4a depicts the characteristics of patients between the two groups. Regarding primary lesions, the incidence of slow growth was significantly higher in the Iq group, whereas that of rapid growth” was significantly higher in the Dq group (*p* < 0.05). The prevalence of pathological fractures, spinal cord compression, and absence of chemotherapy was significantly higher in the Dq group (*p* < 0.001). The proportion of patients with improved NRS max and average scores was significantly higher in the Iq group (*p* < 0.001 and <0.001, respectively).

Furthermore, to explore independent factors associated with improvements in QOL, we performed a multivariate logistic regression analysis (using a stepwise likelihood ratio method). The dependent variable was indicated as 0 for the group with improved QOL and 1 for the group with deteriorated QOL. Primary lesions, as classified by Katagiri, the presence of pathological fractures, spinal cord paralysis, chemotherapy, radiation therapy, changes in NRS scores (average and max), age, and sex were selected as independent variables based on Table 4a. Spinal cord paralysis (OR: 8.416, *p* < 0.001), chemotherapy (OR: 0.249, *p* = 0.007), and NRS average scores (OR: 0.144, *p* < 0.001) were found to significantly influence QOL deterioration (Table 4b).

## 4. Discussion

In this study, we investigated the changes in ADL and QOL of patients with bone metastases who underwent conservative treatment through a BMCB approach. We also examined the factors influencing these changes. The results of the present study showed that patients with bone metastases who underwent conservative treatment through the BMCB exhibited a significant increase in QOL without a decline in ADL. The prevention of SREs, pain management, and improvements in ADL are reported to be important factors associated with improvements in QOL of patients with bone metastases [17]. Hara et al. reported that surgical treatment for bone metastases improved pain and resulted in improvements in ADL and QOL [15]. However, they focused on patients who underwent surgical treatment for bone metastases, and studies on patients who received conservative treatment for bone metastases were not within the scope of our literature review. Patients with primary tumors in the “rapid growth” Katagiri subgroup [18] showed poorer prognosis than patients in other subgroups. Ratasvuori et al. [19] also reported that the type of primary cancer affected the patient’s prognosis. Additionally, in 2004, Hansen et al. [20] reported that lung cancer was associated with poor prognosis. However, advances in drug therapy have enabled some patients with lung cancer to survive longer. It should be noted that the second independent poor prognosis factor, visceral metastasis, also lowers ADL and QOL in patients with cancer. The results of the present study suggest that multidisciplinary treatment for bone metastases may be beneficial, even in cases in which surgical treatment is not performed.

First, we indicated that spinal cord paralysis is one of the factors contributing to the decline in ADL and QOL of patients with bone metastases who received conservative treatment. The progression of spinal metastases leads to spinal cord paralysis and urinary dysfunction, which significantly deteriorates patients’ ADL and QOL [21,22]. Surgical treatment for spinal metastases is effective in preventing spinal cord paralysis. We have previously reported that surgical treatment for spinal metastases improves ADL and QOL and prolongs survival [23,24]. The intervention of the BMCB decreased the number of emergency surgeries for spinal metastases and increased the prevalence of preventive surgical treatments [16]. In the present study, we found that patients with spinal cord paralysis exhibited a decline in ADL and QOL even with multidisciplinary treatments, except surgery. Patients with complete spinal cord paralysis at the time of consultation, which disqualified them for surgery, showed a decline in ADL and QOL. However, had their spinal cord paralysis been prevented earlier, the decline in ADL and QOL could have been prevented. Therefore, early diagnosis and treatment of spinal metastases are crucial to preventing spinal cord paralysis.

Second, the results of the present study revealed the absence of chemotherapy as one of the factors contributing to the decline in ADL and QOL. Notably, several studies have reported that patients with a low PS score did not experience prolonged survival even with chemotherapy [25,26]. The American Society of Clinical Oncology recommends against administering palliative chemotherapy to patients with solid tumors and a PS score of 3–4 but rather advocates for best supportive care [27,28]. Furthermore, patients with bone metastases may have their PS underestimated due to limitations in physical activity from a musculoskeletal perspective. Improved survival prognosis with chemotherapy, even in patients with low PS sores, such as those with small cell lung cancer, has been previously indicated [29]. Furthermore, patients with bone metastasis who are admitted to the rehabilitation ward, where chemotherapy treatment is not feasible, often experience a decline in ADL. Notably, most of these patients are transferred to acute care hospitals for treatment of cancer and complications [30]. Therefore, administering chemotherapy, if possible, might be beneficial for improving the ADL and QOL of patients with bone metastasis, even if their PS score is low.

Finally, we found that inadequate pain control is one of the factors contributing to decreased ADL and QOL. In the management of bone metastases, pain often occurs regardless of the site of metastasis, highlighting the importance of pain control [17]. Furthermore, the results of the present study indicated that the NRS average score, rather than the NRS max score, was a significant risk factor for decreased ADL and QOL. This suggests the importance of controlling persistent pain over sporadic pain.

In the present study, we focused on cases in which multidisciplinary treatment was administered for bone metastases through the BMCB approach. In particular, we analyzed cases determined to be outside surgical indications within the scope of the BMCB. The usefulness of multidisciplinary meetings in the treatment of bone metastasis has been previously suggested [31]. However, in Japan, the proportion of facilities implementing the BMCB is only 16% of cancer care centers and hospitals [32]. Our current findings indicate that the BMCB is useful not only for surgical treatment but also for conservative therapy. Therefore, increasing the number of facilities that implement the BMCB is necessary.

This study has some limitations. First, it was a retrospective and single-arm study. During the study period, the criteria for treatment indications varied, and there might be biases in patient and treatment selection. Second, the patients included in this study had different primary tumors, and the treatment regimens and durations were not standardized. Third, this study did not examine factors such as age, sex, or systemic diseases, e.g., diabetes. In the future, comparative studies with matched patient backgrounds using propensity score matching may be necessary. Finally, we compared changes over a short period in this study, and long-term follow-up was not conducted. Further investigation is needed to assess long-term outcomes, such as overall survival.

## 5. Conclusions

In this study, overall improvement in QOL without a decline in ADL was observed in patients who underwent conservative treatment through a BMCB over a relatively short period. Furthermore, the presence of paralysis, absence of chemotherapy, and poor pain control were found to be associated with a higher risk of deterioration in ADL and QOL.

## Figures and Tables

**Table 1:** (**a**). Baseline characteristics (*n* = 200). (**b**). Primary lesions of bone metastases (*n* = 200).

**(a)**		
**Variables**	***n***	**Total**
Patients		
Age, mean ± SD	200	72.2 ± 12.9
Male sex, *n* (%)	200	116 (58)
Tumor progression		
Primary lesions, as classified by Katagiri, *n* (%)	200	
Slow growth		48 (24)
Moderate growth		71 (35.5)
Rapid growth		81 (40.5)
Other metastasis, *n* (%)	200	
No metastasis		113 (56.5)
Visceral/brain metastasis		77 (38.5)
Dissemination		10 (5.0)
Treatment		
Rehabilitation	200	
Yes		69 (34.5)
No		131 (65.5)
History of chemotherapy, *n* (%)	200	
Yes		118 (59)
No		82 (41)
History of radiotherapy, *n* (%)	200	
Yes		104 (52)
No		96 (48)
Bone-modifying agent		
Yes		79 (39.5)
No		121 (60.5)
ADL (activities of daily living)		
ECOG-PS, *n* (%)	200	
0		19 (9.5)
1		71 (35.5)
2		48 (24)
3		48 (24)
4		14 (7)
Bone metastasis		
Number of bone metastasis, *n* (%)	200	
1		67 (33.5)
≥2		133 (66.5)
Localization of bone metastasis, *n* (%)	200	
Spine		177 (88.5)
Femur and pelvis		54 (27)
Other		42 (21)
SREs, *n* (%)	200	
Fracture		23 (11.5)
Spinal cord paralysis		21 (10.5)
Hypercalcemia		19 (9.5)
(**b**)		
**Primary Lesion**	***n***	**%**
Lung cancer	47	17.3
Renal cell cancer	33	12.1
Head and neck cancer	21	7.7
Breast cancer	17	6.3
Hepatocellular carcinoma	13	4.8
Prostate cancer	9	3.3
Multiple myeloma	9	3.3
Gynecological cancer	8	2.9
Urothelial cancer	8	2.9
Malignant cancer	6	2.2
Colon cancer	5	1.8
Esophageal cancer	4	1.5
Pancreatic cancer	2	0.7
Others	18	6.6

**Table 2:** Changes in assessment scores (mean ± SE).

	Initial Assessment	Reassessment	*p*-Value
PS	1.84 ± 0.08	1.72 ± 0.08	*p* = 0.065
BI	83.15 ± 1.68	84.42 ± 1.73	*p* = 0.054
EQ-5D	0.57 ± 0.02	0.64 ± 0.02	*p* < 0.001
NRS max	5.21 ± 0.24	3.56 ± 0.21	*p* < 0.001
NRS average	2.98 ± 0.18	1.85 ± 0.13	*p* < 0.001

**Table 3:** (**a**) Univariate analysis of factors influencing BI. (**b**) Odds ratios, confidence intervals, and *p*-values of factors influencing ADL deterioration according to multivariate logistic regression analysis.

**(a)**			
**Factor**	**Ia (Increase), *n***	**Da (Decline), *n***	***p*** **-Value**
Age	72.6 ± 12.6	70.9 ± 14.0	0.552
Sex			0.473
Female	68	16	
Male	89	27	
Rehabilitation			0.146
−	113	26	
+	44	17	
Primary lesions, as classified by Katagiri			0.017
Slow growth	43	5	
Moderate growth	58	13	
Rapid growth	56	17	
Pathological fracture			0.029
−	143	34	
+	14	9	
Spinal cord paralysis			<0.001
−	147	32	
+	10	11	
Chemotherapy			0.003
−	84	34	
+	73	9	
Radiotherapy			0.052
−	81	15	
+	76	28	
Multiple bone metastasis			0.108
−	57	10	
+	100	33	
Bone-modifying agent			0.721
−	96	25	
+	61	18	
Change in NRS (Numerical Rating Scale) max score			0.02
Improvement	131	29	
Deterioration	26	14	
Change in NRS average score			0.004
Improvement	134	28	
Deterioration	23	15	
(**b**)			
**Factor**	**OR**	**95% CI**	***p*** **-Value**
Primary lesion classified by Katagiri	1.731	1.049–2.855	0.032
Spinal cord paralysis	3.693	1.370–9.956	0.049
Chemotherapy	0.429	0.184–0.998	0.02
Change of NRS average	0.378	0.166–0.859	0.02

**Table 4:** (**a**) Univariate analysis of factors influencing EQ-5D scores. (**b**) Odds ratios, confidence intervals, and *p*-values of factors influencing QOL deterioration according to multivariate logistic regression analysis.

**(a)**			
**Factor**	**Iq (Increase), n**	**Dq (Decline), n**	***p*** **-Value**
Age	71.8 ± 13.0	74.0 ± 12.3	0.472
Sex			0.052
Female	73	11	
Male	88	28	
Rehabilitation			0.463
−	110	29	
+	51	10	
Primary lesions, as classified by Katagiri			0.022
Slow growth	45	3	
Moderate growth	56	15	
Rapid growth	60	21	
Pathological fracture			<0.001
−	149	28	
+	12	11	
Spinal cord paralysis			<0.001
−	153	26	
+	8	13	
Chemotherapy			<0.001
−	85	33	
+	76	6	
Radiotherapy			0.092
−	82	14	
+	79	25	
Multiple bone metastasis			0.246
−	57	10	
+	104	29	
Bone-modifying agent			0.189
−	101	20	
+	60	19	
Change in NRS max scores			<0.001
Improvement	138	22	
Deterioration	23	17	
Change in NRS average scores			<0.001
Improvement	142	20	
Deterioration	19	19	
(**b**)			
**Factor**	**OR**	**95% CI**	***p*** **-Value**
Spinal cord paralysis	8.416	2.825–25.072	<0.001
Chemotherapy	0.249	0.091–0.683	0.007
Change of NRS average	0.144	0.059–0.347	<0.001

## Data Availability

The data presented in this study are available on request from the corresponding author. The data are not publicly available owing to privacy and ethical concerns.
